# RNA Sequencing Data for FFPE Tumor Blocks Can Be Used for Robust Estimation of Tumor Mutation Burden in Individual Biosamples

**DOI:** 10.3389/fonc.2021.732644

**Published:** 2021-09-28

**Authors:** Maxim Sorokin, Alexander Gorelyshev, Victor Efimov, Evgenia Zotova, Marianna Zolotovskaia, Elizaveta Rabushko, Denis Kuzmin, Alexander Seryakov, Dmitry Kamashev, Xinmin Li, Elena Poddubskaya, Maria Suntsova, Anton Buzdin

**Affiliations:** ^1^ Biostatistics and Bioinformatics Subgroup, European Organization for Research and Treatment of Cancer (EORTC), Brussels, Belgium; ^2^ The Laboratory of Clinical and Genomic Bioinformatics, I.M. Sechenov First Moscow State Medical University, Moscow, Russia; ^3^ Laboratory for Translational Genomic Bioinformatics, Moscow Institute of Physics and Technology, Dolgoprudny, Russia; ^4^ OmicsWay Corp., Walnut, CA, United States; ^5^ Medical Holding SM-Clinic, Moscow, Russia; ^6^ Shemyakin-Ovchinnikov Institute of Bioorganic Chemistry, Russian Academy of Sciences, Moscow, Russia; ^7^ Department of Pathology & Laboratory Medicine, University of California Los Angeles (UCLA) Technology Center for Genomics & Bioinformatics, Los Angeles, CA, United States; ^8^ World-Class Research Center “Digital Biodesign and Personalized Healthcare”, Sechenov First Moscow State Medical University, Moscow, Russia

**Keywords:** TMB (tumor mutation burden), RNAseq, FFPE (formalin-fixed paraffin-embedded), machine learning, oncology

## Abstract

Tumor mutation burden (TMB) is a well-known efficacy predictor for checkpoint inhibitor immunotherapies. Currently, TMB assessment relies on DNA sequencing data. Gene expression profiling by RNA sequencing (RNAseq) is another type of analysis that can inform clinical decision-making and including TMB estimation may strongly benefit this approach, especially for the formalin-fixed, paraffin-embedded (FFPE) tissue samples. Here, we for the first time compared TMB levels deduced from whole exome sequencing (WES) and RNAseq profiles of the same FFPE biosamples in single-sample mode. We took TCGA project data with mean sequencing depth 23 million gene-mapped reads (MGMRs) and found 0.46 (Pearson)–0.59 (Spearman) correlation with standard mutation calling pipelines. This was converted into low (<10) and high (>10) TMB per megabase classifier with area under the curve (AUC) 0.757, and application of machine learning increased AUC till 0.854. We then compared 73 experimental pairs of WES and RNAseq profiles with lower (mean 11 MGMRs) and higher (mean 68 MGMRs) RNA sequencing depths. For higher depth, we observed ~1 AUC for the high/low TMB classifier and 0.85 (Pearson)–0.95 (Spearman) correlation with standard mutation calling pipelines. For the lower depth, the AUC was below the high-quality threshold of 0.7. Thus, we conclude that using RNA sequencing of tumor materials from FFPE blocks with enough coverage can afford for high-quality discrimination of tumors with high and low TMB levels in a single-sample mode.

## Introduction

Tumor mutation burden (TMB) per million base pairs is a well-known efficacy predictor for checkpoint inhibitor immunotherapy (1). TMB can be calculated in several ways (2). For example, in commercial FDA-approved FoundationOne CDx test for unpaired single tumor samples, TMB is defined as the number of somatic mutations per million base pairs (megabase) of the protein-coding sequence analyzed—including both substitutions and indels, but irrespective of the functional consequences of the variants (3). Highly mutated tumors are more likely to produce tumor neoantigens and become more “visible” to the immune system; thus, TMB is a good proxy for the tumor neoantigen load (4).

To date, TMB assessment is commercially available in the form of clinical and research use-only diagnostic tests (5). In June 2020, TMB was approved for the use of immune checkpoint inhibitor pembrolizumab in the treatment of patients with advanced or metastatic solid cancers, and FoundationOne CDx assay was approved as a companion diagnostic test.

The two major approaches for evaluating TMB are based on using whole exome sequencing (WES) and next-generation sequencing (NGS) panels. WES-TMB was demonstrated first to be associated with tumor responses on immune checkpoint inhibitors and thus proposed as a predictive biomarker (6–9). These early WES-TMB estimates were considering only non-synonymous somatic mutations. Overall, TMB levels were classified as “high” or “low.” However, the cutoff values varied from ≥7.4 in esophageal and gastric cancer till ≥23.1 in non-small cell lung cancer (NSCLC) for the number of mutations per megabase DNA and from ≥158 mutations in advanced NSCLC till ≥248 mutations in advanced small cell lung cancer for the whole tumor exome non-synonymous mutation estimates (10).

To address many of the WES-TMB limitations, targeted sequencing panels with exonic sequences of especially frequently mutated genes were developed to estimate TMB (11, 12). Unlike in WES-TMB, NGS panels count both non-synonymous and synonymous mutations as well as indels, which can increase assay sensitivity (3). This approach showed that sufficiently large NGS panels can accurately recapitulate WES-TMB, and demonstrated good agreement between panels-derived and WES-derived TMB values (13, 14).

There are two NGS panels commercially available to date that have been approved by regulatory bodies: FoundationOne CDx assay approved by the FDA as a companion diagnosis for the assessment of TMB and the MSK-IMPACT panel.

Overall, many variable factors can influence TMB estimation and output, including tumor type (15), biosample type (FFPE materials artificially have more mutations than fresh frozen tissue), and sequencing parameters (NGS panel content, size, and sequencing depth; bioinformatic pipeline; and reporting cutoff) (16).

At the moment, DNA analysis is the only standard for TMB assessment and it is largely unclear whether TMB derived from RNA sequencing (RNAseq) corresponds to DNAseq data. In 2020, Jang and coauthors attempted to calculate TMB from single-cell RNA sequencing data (17). However, the authors did not provide any technical rationale for their approach and did not validate it by DNA mutation analysis, thus leaving the adequacy of the results communicated uncertain. In February 2021, DiGuardo and colleagues demonstrated a correlation between RNAseq- and DNAseq-derived TMB using formalin-fixed, paraffin-embedded (FFPE) tumor tissue blocks (18). However, this was more a proof-of-concept study done for only eight individual samples and the methodological limitations of this approach were not explored. Furthermore, this was done for the matched pairs of tumors and adjacent normal tissues, while matching healthy samples are not frequently available in the routine clinical practice. For example, the FoundationOne CDx test utilizes single tumor-only biosamples to return TMB (2).

FFPE cancer tissue biosamples are known to yield highly fragmented nucleic acid preparations that could be hardly applicable to the tasks requiring high RNA integrity like analysis of differential splicing (19). Nucleic acids extracted from FFPE also often contain artifact alterations caused by formalin fixation, thus having an increased rate of C>T substitutions, when compared with nucleic acids from fresh tissues. Thus, sequencing profiles derived from FFPE should be processed differently and the results obtained from fresh tissues may be poorly compatible. However, FFPE materials can be used to properly estimate TMB by the DNA screens (20) and for the clinical-grade estimation of the gene expression levels by analyzing RNA reads (21, 22). From certain points of view, this is the preferred type of biomaterial because of its high availability and stability, as FFPE blocks can be stored at room temperature for years prior to nucleic acid extraction for sequencing purposes (23).

Thus, in this study, we investigated whether FFPE-isolated RNA can be used for TMB estimates without the analysis of adjacent or blood-derived norms. To this end, we used the paired RNAseq–WES profiles for FFPE materials available from The Cancer Genome Atlas (TCGA) project database, in both paired and single-sample modes, and then validated the results using 73 experimental RNAseq–WES profiles obtained for FFPE cancer specimens.

For TMB deduced using TCGA RNAseq data, where mean sequencing depth was ~23 million gene-mapped reads (MGMRs), we obtained 0.46 (Pearson)–0.59 (Spearman) correlation with the standard mutation calling pipelines. This was converted in the classifier of low (<10) and high (>10) TMB per megabase with area under the curve (AUC) 0.757, and the application of machine learning (ML) increased AUC till 0.854. We then compared 73 experimental pairs of WES and RNAseq profiles with lower (mean 11 MGMRs) and higher (mean 68 MGMRs) sequencing depths. We observed 0.85 (Pearson)–0.95 (Spearman) correlation of TMB with standard mutation calling pipelines for higher RNA sequencing depth samples, and ~1 AUC for the high/low TMB classifier. However, for the lower depth, the AUC was below the high-quality threshold of 0.7 even in case of applying ML. Thus, we conclude that using RNA sequencing of tumor materials from FFPE blocks with enough coverage can afford for high-quality discrimination of high and low TMB tumors in a single-sample mode.

## Materials and Methods

### Reference Public Dataset

The available set of matching tumor WES/FFPE RNAseq and normal (blood or adjacent non-cancerous tissue) WES FASTQ files corresponding to the same tumors, a total of 53 samples, was downloaded from The Cancer Genome Atlas (TCGA) international project repository (https://portal.gdc.cancer.gov/), and only FFPE samples of primary tumors were selected (**Table S1**).

### Experimental Tissue Samples

All experimental data were obtained for pathologist-verified FFPE tumor tissue blocks with tumor cell content greater than 50%. The sample annotation contained information about the sex, age, and cancer type of the patient (**Table S2**). In all cases, written informed consents to participate in this study were acquired from the patients or from their legal representatives. The consent procedure and the design of the study were approved by the ethical committees of the Karelia Republic Oncological Hospital, Petrozavodsk, Russia, and Vitamed Oncological Clinical Center, Moscow, Russia.

### RNAseq: Library Preparation and Sequencing

RNA sequencing was performed according to the previous protocol used to generate ANTE collection of healthy tissue RNAseq profiles (23) and several cancer expression collections (22, 24–28). To isolate RNA preps, 10-μM-thick paraffin slices were trimmed from each FFPE tissue block with a microtome. RNA was extracted from FFPE slices using Qiagen RNeasy FFPE kit following the protocol of the manufacturer. RNA 6000 Nano or Qubit RNA Assay kits were used to measure RNA concentration. RNA integrity number (RIN) was measured using Agilent 2100 Bioanalyzer. For depletion of ribosomal RNA and library construction, KAPA RNA Hyper with rRNA erase kit (HMR only) was used. Different adaptors were used for multiplexing samples in a single sequencing run. Library concentrations were measured using the Qubit dsDNA HS Assay kit (Life Technologies) and Agilent TapeStation (Agilent). RNA sequencing was performed at the Department of Pathology and Laboratory Medicine, University of California Los Angeles, using Illumina HiSeq 3000 equipment for single-end sequencing, 50 bp read length, achieving a median of ~217 million raw reads or ~68 million mapped reads per sample. Illumina SAV was used for data quality checks. De-multiplexing was performed with Illumina Bcl2fastq2 Conversion Software v2.17.

Sequencing data were deposited in NCBI Sequencing Read Archive (SRA) under accession ID PRJNA733593.

### Whole Exome Sequencing: Library Preparation and Sequencing

DNA WES was performed according to (29). DNA was extracted from the FFPE tissue using the AnaPrep FFPE DNA extraction kit and whole exome DNA was captured from total genomic DNA using the SeqCap EZ System from NimbleGen according to the instructions of the manufacturer. Briefly, genomic DNA was sheared, size selected to roughly 200–250 base pairs, and the ends were repaired and ligated to specific adapters and multiplexing indexes. Fragments were then incubated with SeqCap biotinylated DNA baits followed by the LM-PCR, and the RNA–DNA hybrids were purified using streptavidin-coated magnetic beads. The RNA baits were then digested to release the targeted DNA fragments, followed by a brief amplification of 15 or less PCR cycles. Sequencing was performed on Illumina HiSeq 3000 for a pair read 150 run. Data quality check was done on Illumina SAV. Demultiplexing was performed with Illumina Bcl2fastq2 v 2.17 program.

### Processing of RNA Sequencing Data

**Figure 1:**
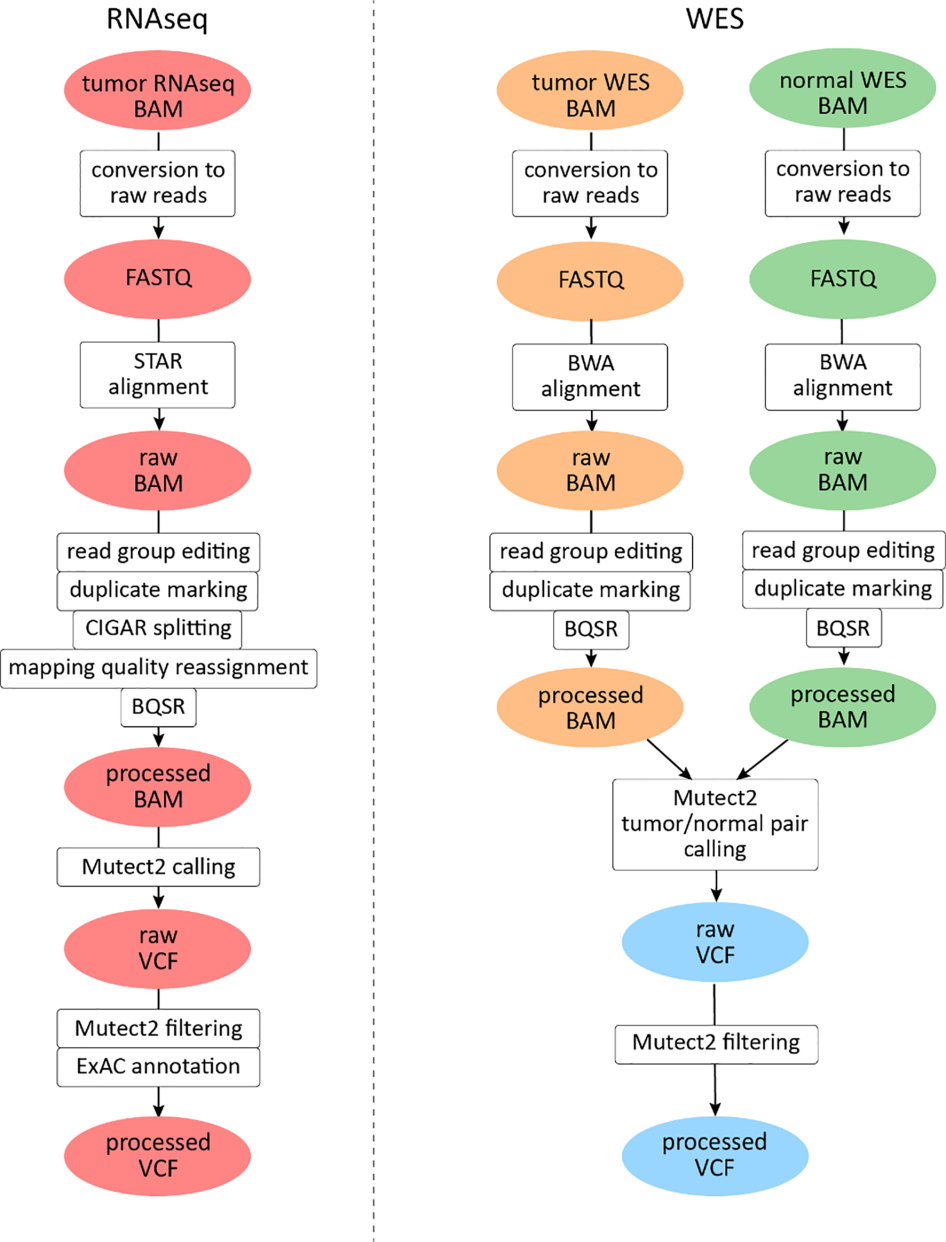
Mutation calling pipeline for The Cancer Genome Atlas (TCGA) data analysis. File instances are shown in ovals, pipeline steps in rectangles. RNAseq files are highlighted in red, tumor whole exome sequencing (WES) files in orange, and normal WES files in green. Data derived from both tumor and normal WES data are shown in light blue.

For RNAseq data, a GATK mutation calling pipeline was used (**Figure 1**) (30). Reads were aligned to the human genome assembly GRCh38 with STAR v2.6.1d software in two-pass mode (31). The following parameters were set to non-default values: sjdbOverhang 100, twopass1readsN 10000000, and twopassMode Basic.

Exon coordinates were taken from Ensembl annotation version 89. Samtools v1.3.1 package was used for BAM file indexing (32). All reads were assigned to a single read group, and read group information editing and duplicate marking were performed with AddOrReplaceReadGroups and MarkDuplicates software, respectively (http://broadinstitute.github.io/picard). GATK v3.8.0 SplitNCigarReads module was used to split reads that aligned to exon junctions. Base quality score recalibration was performed with GATK v4.beta.1 BaseRecalibrator and ApplyBQSR modules. For mutation calling, GATK4 Mutect2 software (33) was used in tumor-only mode with dbSNP version 146 variant database (34) and 1000G gold standard indel database (35). No panel of norms was used for the experimental settings.

Variants were called only in exonic regions of human chromosomes 1–22, X, and Y (mapped according to GENCODE (36), and the PCR indel model parameter was set to “HOSTILE”. Variants were filtered with GATK4 FilterMutectCalls (all variants are kept in VCF, only the “FILTER” field is edited). Predicted functional effects of variants identified were annotated using ANNOVAR software (37). For variant annotation, we used the version of ExAC database, which does not contain TCGA samples (38). All tri- or more allelic sites were excluded from further analyses: such mutations were not annotated using ANNOVAR and were not included in TMB calculation.

### Processing of WES Data

For WES data analysis, a GATK somatic mutation calling pipeline was used (**Figure 1**). Reads were aligned to the human genome version 38 with BWA mem v0.7.17 software (39). The following parameters were set to non-default values: −k 15, −r 2. The rest of the pre-processing steps were identical to the RNAseq pipeline described above, except for reads splitting and mapping quality editing steps which were skipped.

For mutation calling, GATK4 Mutect2 (33) software was used simultaneously for tumor and matched normal samples, supplied with the same dbSNP and indel databases, regions, and PCR model. Subsequent post-processing steps included filtering GATK4 FilterMutectCalls and annotation with ANNOVAR. All tri- or more allelic sites were excluded from further analyses: such mutations were not annotated using ANNOVAR and were not included in TMB calculation. For parallel computational task management, GNU parallel software was used (40).

### Supervised Machine Learning

For filtering with supervised learning, we selected 31 variant features such as reference allele depth, median base quality, or the number of events in the haplotype. From them, 23 features were taken directly from Mutect2 output VCF files and one was obtained from ANNOVAR annotation of VCF with non-TCGA ExAC database. The other seven features were engineered using data from Mutect2 output VCF: four Boolean features of variant being *i*) an insertion, *ii*) a deletion, *iii*) a C>T (G>A) transition, and *iv*) C>A (G>T) transversion were constructed based on values in REF and ALT fields and three integer features: *v*) total depth and *vi–vii*) REF and ALT lengths were constructed based on values in REF, ALT fields, and FORMAT AD field.

Model hyperparameters were selected during a series of randomized grid searches. Parameters adjusted were as follows: learning_rate, n_estimators, min_child_weight, gamma, subsample, colsample_bytree, max_depth, reg_alpha, and reg_lambda; among them, the first two were the most impactful. Cross-validation was five-fold, and receiver operating characteristic (ROC) AUC was used as the metric for hyperparameter selection. The Python code used for ML and visualization is available at https://gitlab.com/oncobox/tmb_rnaseq.

## Results

To initially explore the possibility of estimating TMB from FFPE RNAseq data, we used a set with matched WES/RNAseq tumor profiles and, at the same time, with the normal control WES data available from the TCGA project database. Totally, data could be obtained for only 53 tumor cases (paired RNAseq and WES data for tumor samples, and WES data for the matched controls) because the absolute majority of the paired TCGA data were generated for fresh-frozen tissue samples.

We then assessed how different RNAseq data filtering options, including ML, can alter the correlation between TMB-RNAseq and TMB-WES. To this end, we randomly assigned 53 available TCGA samples to the training (*n* = 27) and validation (*n* = 26) subsets.

### Modeling of WES-RNAseq-TMB Correlation on TCGA Dataset With Matched Normal WES Controls

**Figure 2:**
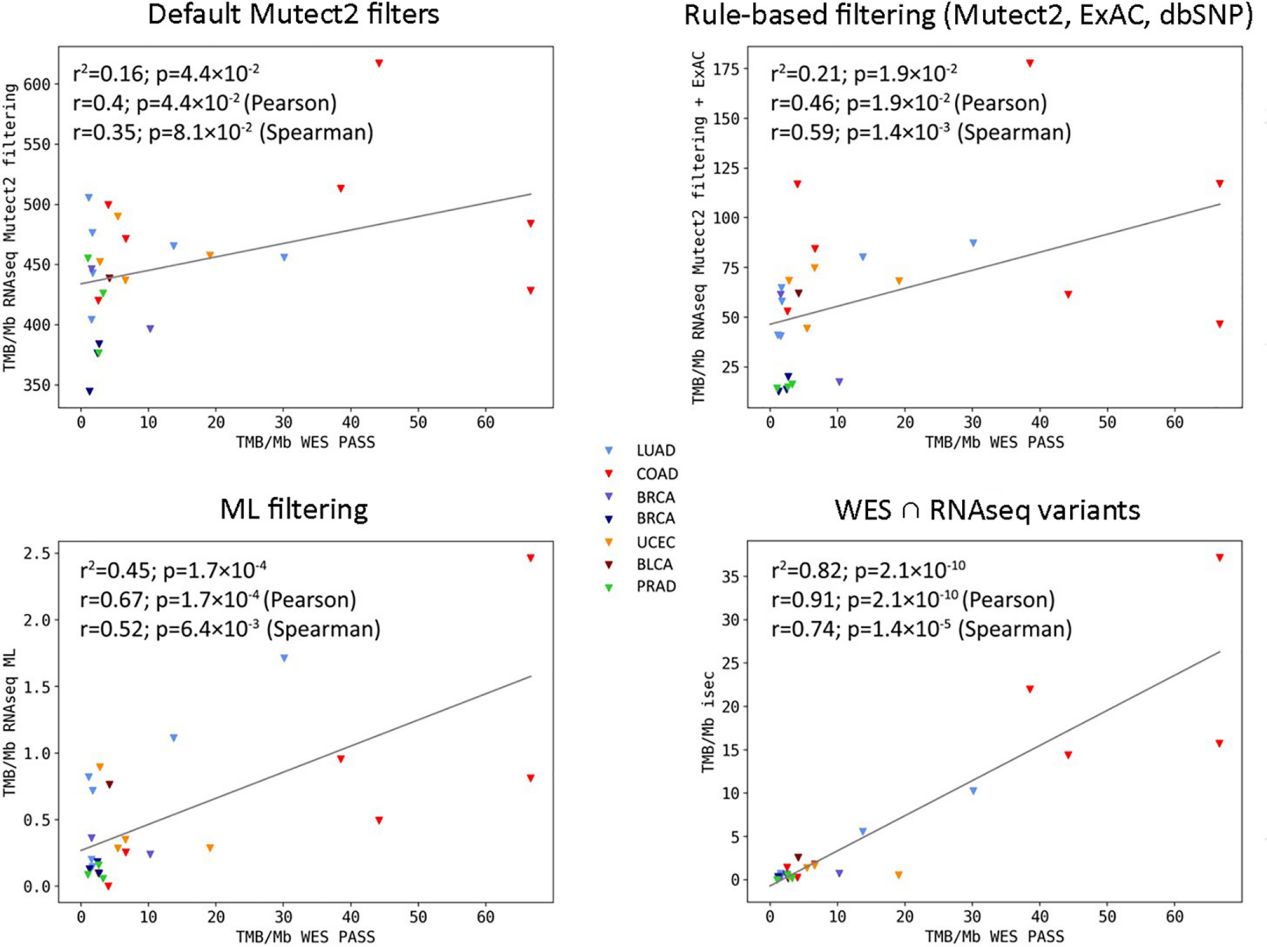
Correlations between RNAseq- and WES-derived tumor mutation burden (TMB) estimates (TCGA FFPE dataset, *n* = 26), with matched normal WES references. Samples are colored according to cancer type. Cancer type abbreviations used: LUAD, lung adenocarcinoma; COAD, colon adenocarcinoma; BRCA, breast invasive carcinoma; UCEC, uterine corpus endometrial carcinoma; BLCA, urothelial bladder carcinoma; PRAD, prostate adenocarcinoma.

In this application, WES tumor mutation calling was performed while taking into account the available WES profiles for the healthy control biosamples from the same patients. Our first step was to determine the biggest possible correlation between RNAseq and WES-TMB estimates for the above FFPE TCGA dataset. To that end, we collected variants common to the WES and RNAseq callsets, as identified by genomic coordinate, reference, and alternative allele. We used variants common to both WES and RNAseq only to estimate maximal possible correlation between TMB derived from WES *vs.* TMB derived from RNAseq (“isec”). For further rule- and ML-based filtering of RNAseq variants presented below, all RNAseq variants were used regardless of their presence in WES data. The WES variants were filtered by the condition “FILTER==PASS,” while RNAseq variants were left unfiltered. The Pearson correlation coefficient between TMB-RNAseq and TMB-WES estimates calculated for the *n* = 206 subset was 0.91, *p* = 2.1 × 10^−10^ (**Figure 2**, “isec”). No discernable sequencing batch bias was detected during both correlation analysis and primary component analysis: samples from different TCGA sequencing centers were randomly clustered on the PCA (**Figure S1**).

We then built the ROC curve and calculated the AUC metric for it. ROC AUC is widely used to assess the performance of biomarkers in oncology (41–43), and it depends on their sensitivity and specificity (44). It varies between 0.5 and 1, and the robustness criterion of biomarkers is typically AUC greater than 0.7 (45).

We selected three clinically relevant thresholds of TMB per megabase: of TMB ≥6, ≥10, and ≥20 (11, 46). Among these, the cutoff value of 10 is currently especially frequently used in clinical studies, although no formal consensus has been reached yet (10). Seven out of 26 samples (27%) had TMB ≥10 Mut/Mb. This is higher than in previous studies investigating pan-cancer patient cohorts with different solid tumors (47–49). Considering WES-derived TMB as the gold standard among the available TMB data, we obtained the AUC scores for TMB-RNAseq of 0.925, 0.903, and 1 for the abovementioned thresholds, respectively (**Table 1**). Note that these estimates considered only the fraction of genome that was sufficiently covered by both WES and RNAseq reads and left apart other sequences that were covered by the RNAseq or WES reads separately.

**Table 1:** ROC AUC scores for predicting TMB (≥6, ≥10, and ≥20 thresholds) from FFPE RNAseq data, according to TMB-WES standards calculated with matched normal WES profiles.

Filtering method	TMB ≥ 6	TMB ≥ 10	TMB ≥ 20
TCGA (matched norm was used for WES mutation calling)			
WES ∩ RNAseq variants	0.925	0.903	1
Default Mutect2 filtering	0.694	0.701	0.781
Default Mutect2 filtering + ExAC <0.000033	0.825	0.757	0.81
ML filtering using XGBoost method	0.825	0.854	0.905
TCGA (matched norm was not used for WES mutation calling)			
WES ∩ RNAseq variants	0.953	0.921	1
Default Mutect2 filtering	0.573	0.64	0.726
Default Mutect2 filtering + ExAC <0.000033	0.693	0.763	0.857
ML filtering using XGBoost method	0.7	0.868	0.857
Experimental dataset (high coverage)			
WES ∩ RNAseq variants	0.75	1	NA
Default Mutect2 filtering	0.812	0.857	NA
Default Mutect2 filtering + ExAC <0.000033	1	1	NA
Experimental dataset (low coverage)			
WES ∩ RNAseq variants	0.719	0.748	NA
Default Mutect2 filtering	0.407	0.226	NA
Default Mutect2 filtering + ExAC <0.000033	0.622	0.674	NA
ML filtering using XGBoost method	0.53	0.613	NA

NA, Not applicable.

We then simulated a situation when there are no available WES data for matched healthy controls. To this end, we assessed the performance of algorithmic RNAseq data filtering, treating it as a baseline. The selected variants used for TMB calculations were *i*) marked as “germline_risk” or “panel_of_normals, germline_risk” by Mutect2, *ii*) had ExAC frequency <0.000033 (3), and *iii*) had no associated dbSNP150 identifier. Mutect2 software filters were chosen here to accommodate for the lack of paired normal samples for RNAseq data. Using this approach, we observed a weak yet statistically significant correlation between the TMB-RNAseq and TMB-WES estimates with Pearson correlation 0.46, *p* = 0.019 (**Figure 2B**), suggesting the need for a more advanced filtering method.

**Figure 3:**
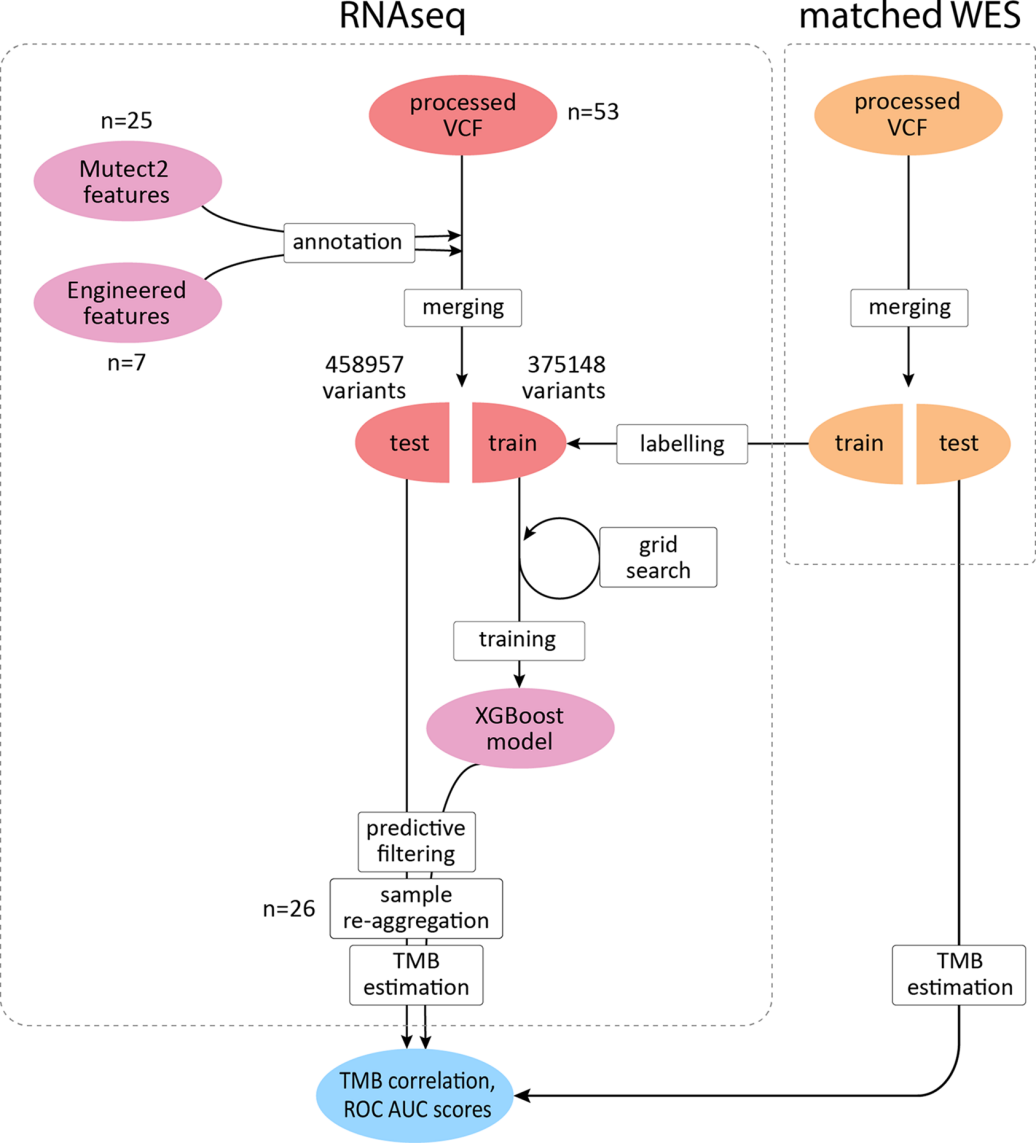
XGBoost binary classifier development workflow. TCGA FFPE samples with matched WES and RNAseq data were reanalyzed to produce callsets in the VCF format. Thirty-two features were introduced to the model in total. RNAseq files along with matching WES files were randomly assigned to two subgroups with variants merged respectively to obtain two sets of variants for each data source. RNAseq variants from the training subgroup were labeled by cross-referencing with the WES callset. Variants matched in WES callset by genomic coordinate were labeled as “signal” and the rest as “noise.” After the model was trained to distinguish between the two classes and validated, variants in the testing subset were reaggregated per sample. Filtering out variants predicted as “noise,” testing per-sample callsets were used to calculate TMB estimates and compared against the respective WES-derived estimates to obtain correlation coefficients and ROC AUC scores.

To improve the correlation and decrease the signal-to-noise ratio, we developed a supervised ML binary classifier, based on the XGBoost algorithm (50) (**Figure 3**). The *n* = 27 subset (458,957 variants called) was used here as a training dataset, and the *n* = 26 subset (375,148 variants called) as a test subset. Prior to the analysis, unfiltered RNAseq VCF files were merged within each subset. Each variant in the training subset was labeled as either “signal” or “noise” based on whether a variant with the same genomic coordinate was discovered in the accompanying WES “gold standard” data. Confusion matrices for the ML model predictions on the training and testing subsets are shown in **Table 2**, and feature importance scores (gain), assigned by the ML model, are shown in **Figure S2**. These scores reflect the value of each feature in the construction of the boosted decision trees within the model. The more an attribute is used to make key decisions for the trees, the higher is its relative importance.

**Table 2:** Confusion matrices for the XGBoost binary classifier predictions (TCGA FFPE dataset, according to WES-TMB standards calculated with matched normal WES profiles).

	No WES variant	WES variant	
A. Training dataset	457,276 (TN)	1,050 (FN)	Predicted noise
	60 (FP)	571 (TP)	Predicted signal
B. Testing dataset	373,731 (TN)	1,218 (FN)	Predicted noise
	101 (FP)	98 (TP)	Predicted signal

The top 5 features that obtained the highest scores by the ML model were as follows: *i*) ExAC_nontcga_ALL (allele frequency as observed in the complete ExAC database, excluding only participants from the TCGA project), *ii*) SA_MAP_AF_2 (the maximum likelihood estimate of the allele fraction given no artifacts on either strand), *iii*) INS (whether or not the variant is an insertion), *iv*) TLOD (log-odds that the variant is present in the tumor sample relative to the expected noise), and *v*) SA_MAP_AF_1 (the maximum likelihood estimate of the allele fraction given an artifact on the reverse strand). SA_MAP_AF_1/2 parameters are used for TLOD calculation, which, in turn, together with ExAC allele frequency, were used for rule-based filtering. Thus, ML outperforms rule-based filtering possibly due to more optimal thresholds (or their combinations) for these parameters. Whether the variant is an insertion or not was not included into the rule-based filtering; however, according to the model used, this might be an important parameter for selecting true variants. The latter may be due to possible bias of insertions between true and false variants. Other features considered by the ML model built are shown in **Table S3**.

**Figure 4:**
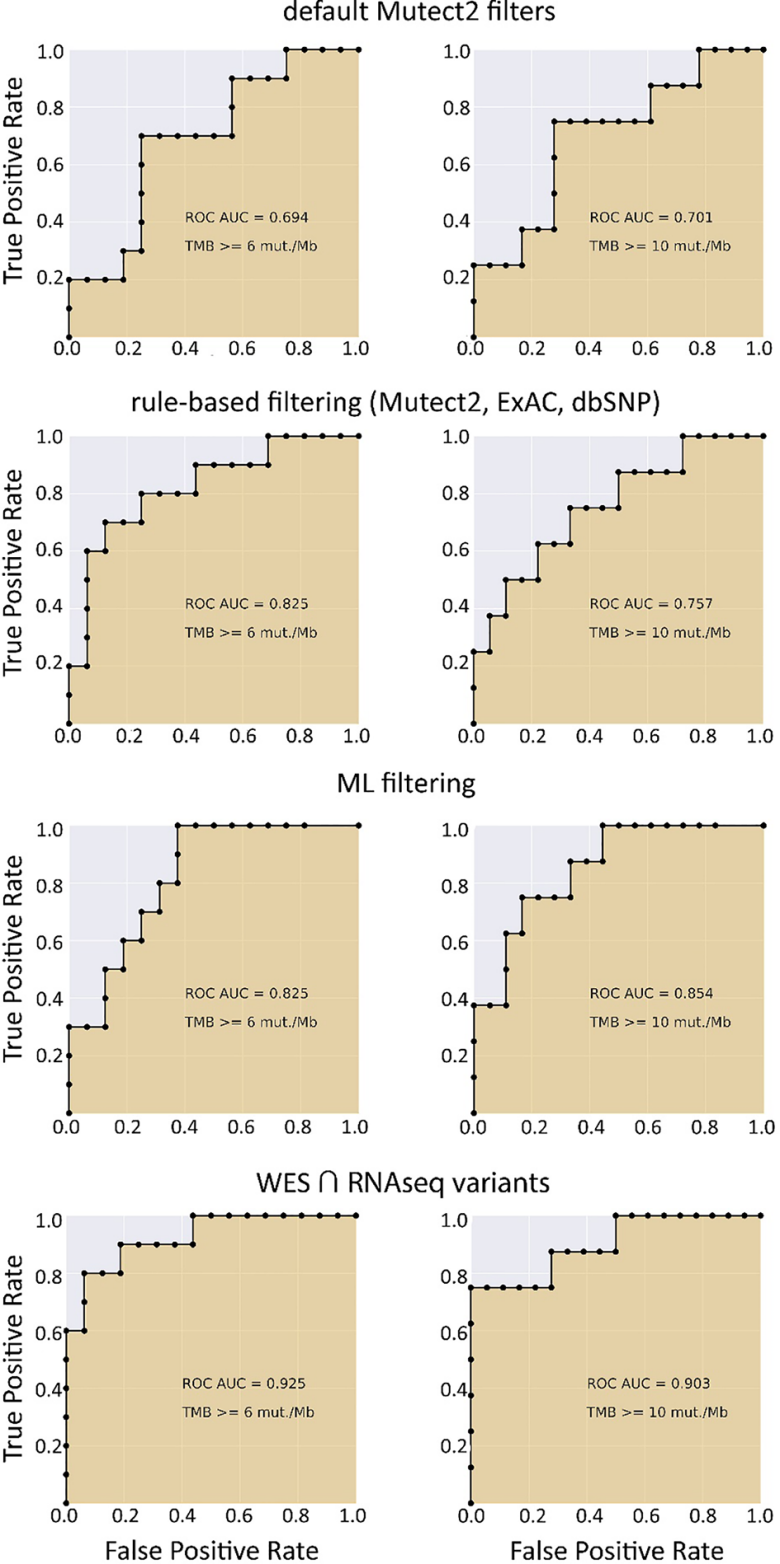
ROC AUC scores for predicting WES-TMB per megabase with RNAseq-TMB per megabase (TCGA FFPE dataset, *n* = 26).

When applying data filtering with the ML model obtained, we observed on the test subset a significantly better Pearson correlation between TMB-RNAseq and TMB-WES of 0.67, *p* = 1.7 × 10^−4^ (**Figure 2C**). Importantly, these results were obtained despite significant sample-to-sample variability in precision and universally low recall (**Figure S3**). The ROC AUC values obtained were also relatively high: 0.825, 0.854, and 0.905 for TMB thresholds of ≥6, ≥10, and ≥20 mutations per megabase, respectively (**Figure 4** and **Table 1**).

### Modeling of WES-RNAseq-TMB Correlation on TCGA Dataset Without Matched Normal WES Controls

We then tested whether RNAseq-TMB data are congruent with the WES-TMB data when no matched healthy tissue TBM profile is available. To this end, we took the same *n* = 26 and *n* = 27 subsets of 53 TCGA profiles. The difference was that the matched healthy WES profiles were not used when calculating the “gold standard” WES-TMB values for tumor samples. The data processing workflow was modified accordingly. First, the following specific criteria were added to WES variant filtering: *i*) annotated ExAC ALL frequency <0.000033 and *ii*) no associated dbSNP150 identifier. These filtering rules were chosen to help discern between germline and somatic variants in the absence of paired WES norms.

**Figure 5:**
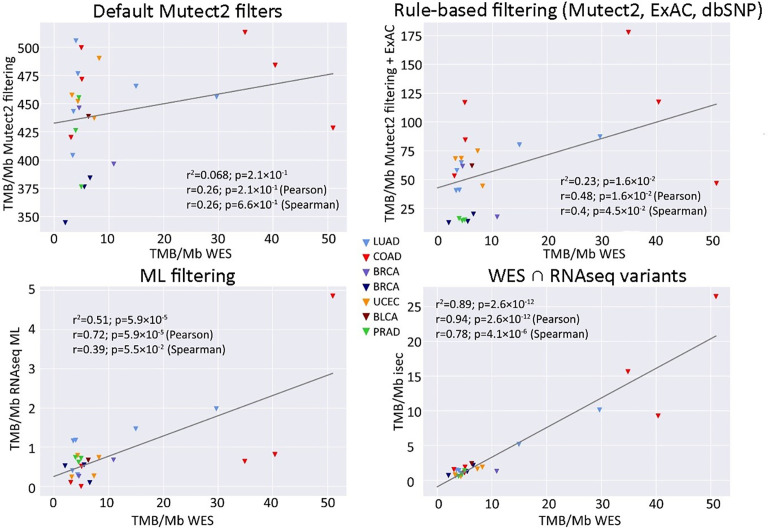
Correlations between RNAseq- and WES-derived TMB estimates (TCGA FFPE dataset, *n* = 26), without matched normal WES references. Samples are colored according to cancer type. Cancer type abbreviations used: LUAD, lung adenocarcinoma; COAD, colon adenocarcinoma; BRCA, breast invasive carcinoma; UCEC, uterine corpus endometrial carcinoma; BLCA, urothelial bladder carcinoma; PRAD, prostate adenocarcinoma.

**Figure 6:**
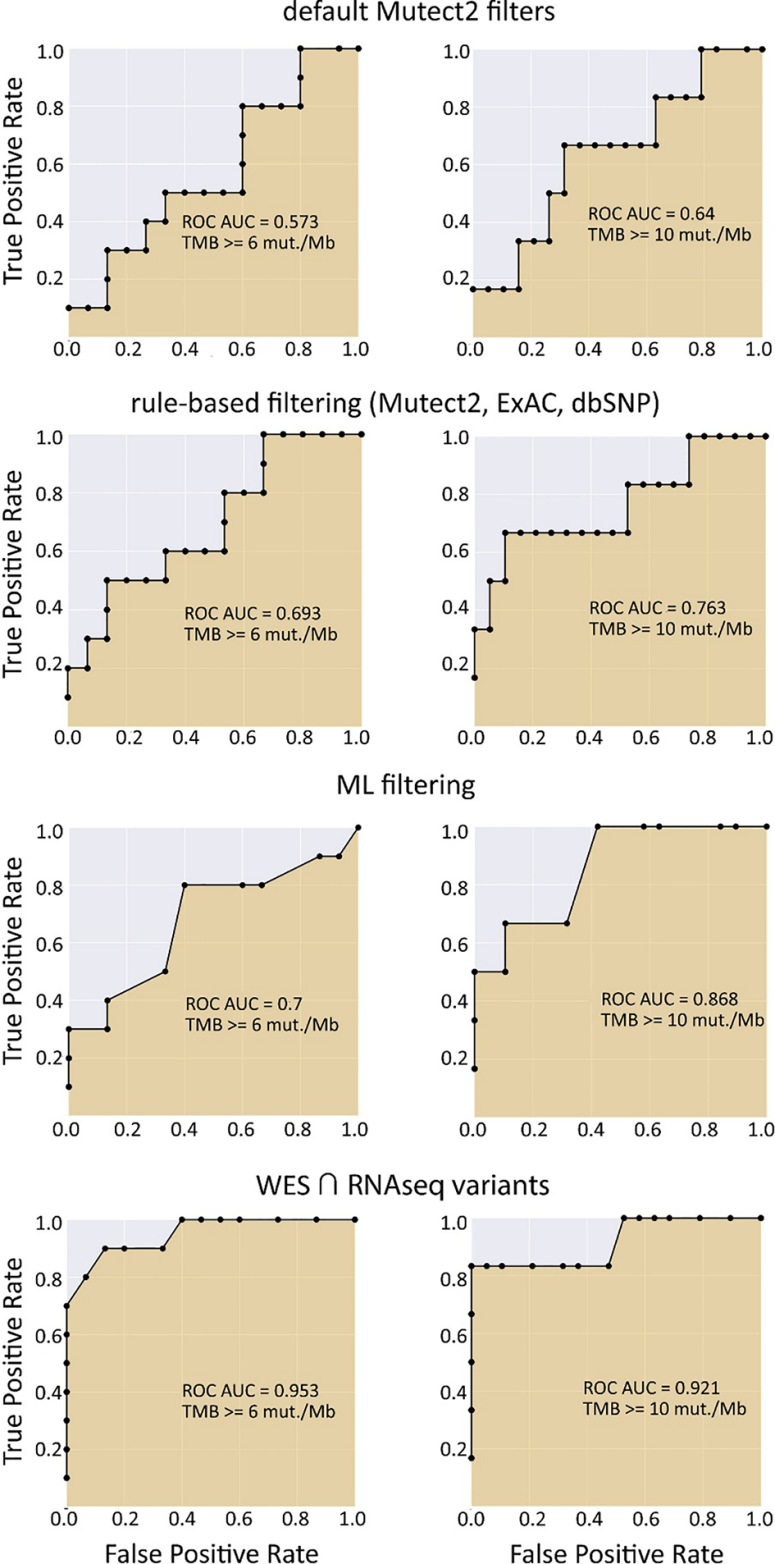
ROC AUC scores for predicting WES-TMB per megabase with RNAseq-TMB per megabase (TCGA FFPE dataset, *n* = 26, no matched norm was used for mutation calling in WES).

We observed that algorithmic filtering of RNAseq data resulted in a modest, yet significant correlation between RNAseq-TMB and WES-TMB data: Pearson correlation 0.48, *p* = 0.016 (**Figure 5**). The ROC AUC values obtained were 0.693, 0.763, and 0.857 for TMB thresholds of ≥6, ≥10, and ≥20, respectively (**Figure 6** and **Table 1**).

In turn, using XGBoost ML filtering allowed to increase this correlation up to 0.72 (*p* = 5.9 × 10^−5^), while the theoretically deduced maximum possible correlation was 0.94 (*p* = 2.6 × 10^−12^, **Figure 5**). In the case of ML filtering, the ROC AUC values increased up to 0.7 and 0.868 for TMB thresholds of ≥6 and ≥10, respectively, while ROC AUC for the threshold of ≥20 did not change (0.857). ML-based prediction for ≥10 TMB threshold was very close to the possible maximum (AUC 0.87 *vs.* 0.92).

Thus, we conclude that using RNAseq-based TMB assessment in one-sample mode can be robust for all three TMB thresholds of ≥6, ≥10, and ≥20, as evidenced by the TCGA quality-paired WES/RNAseq dataset.

### Experimental Evaluation of RNAseq-TMB in Comparison With WES-TMB

**Figure 7:**
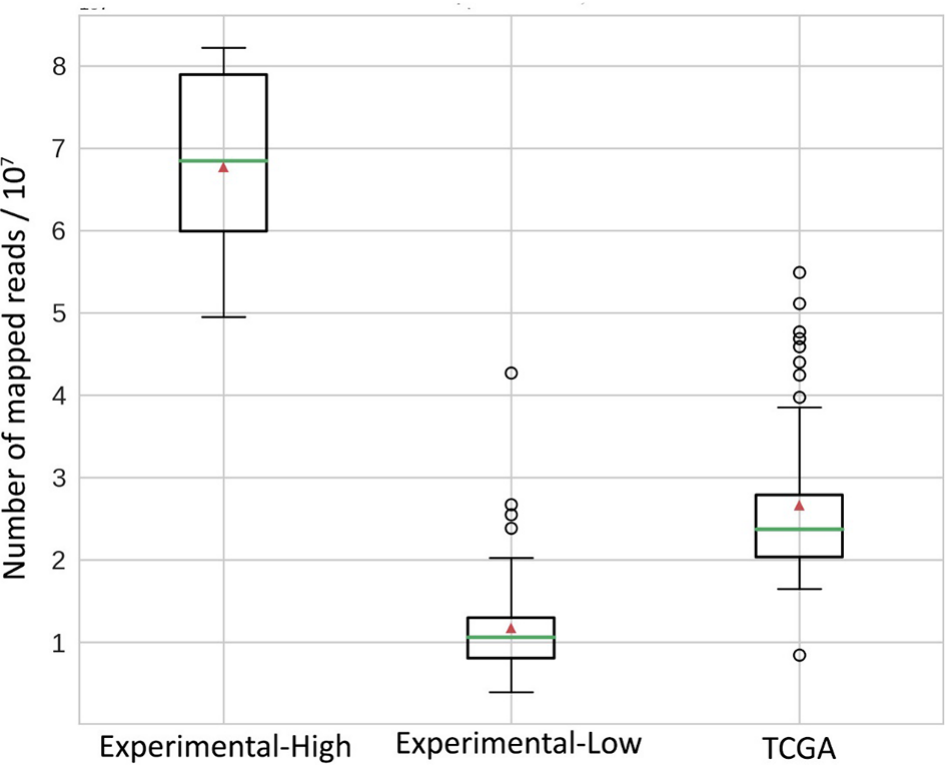
Distribution of mapped reads in RNAseq BAM files. All reads were approximately 50 bases long. Green horizontal lines indicate the median, red triangles indicate the mean.

The easiest diagnostic solution would ideally comprise analysis of just one tumor sample. We found on the previous step with the TCGA model dataset that RNAseq-TMB estimates can afford for robust discrimination between the high and low TMB groups even without matched healthy controls. We hypothesized that RNAseq-TMB estimates may depend on the RNA sequencing coverage. To test this hypothesis, we did experimental paired RNAseq and WES sequencing for 73 FFPE solid tumor tissue biosamples from different cancer patients (**Table S3**). Among them, for 65 samples, we aimed to obtain RNAseq profiles with ~2.5 times lower coverage than in the model TCGA dataset, and for 8 samples, with ~2.5 times higher coverage (**Figure 7**).

### TMB Evaluation for Experimental RNAseq Data With “Low” Coverage

**Figure 8:**
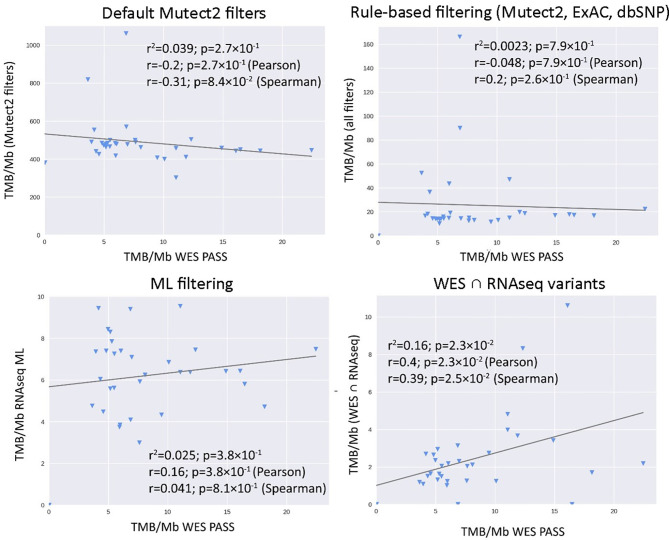
Correlations between RNAseq- and WES-derived tumor mutation burden estimates in experimental low coverage FFPE dataset, *n* = 31.

**Figure 9:**
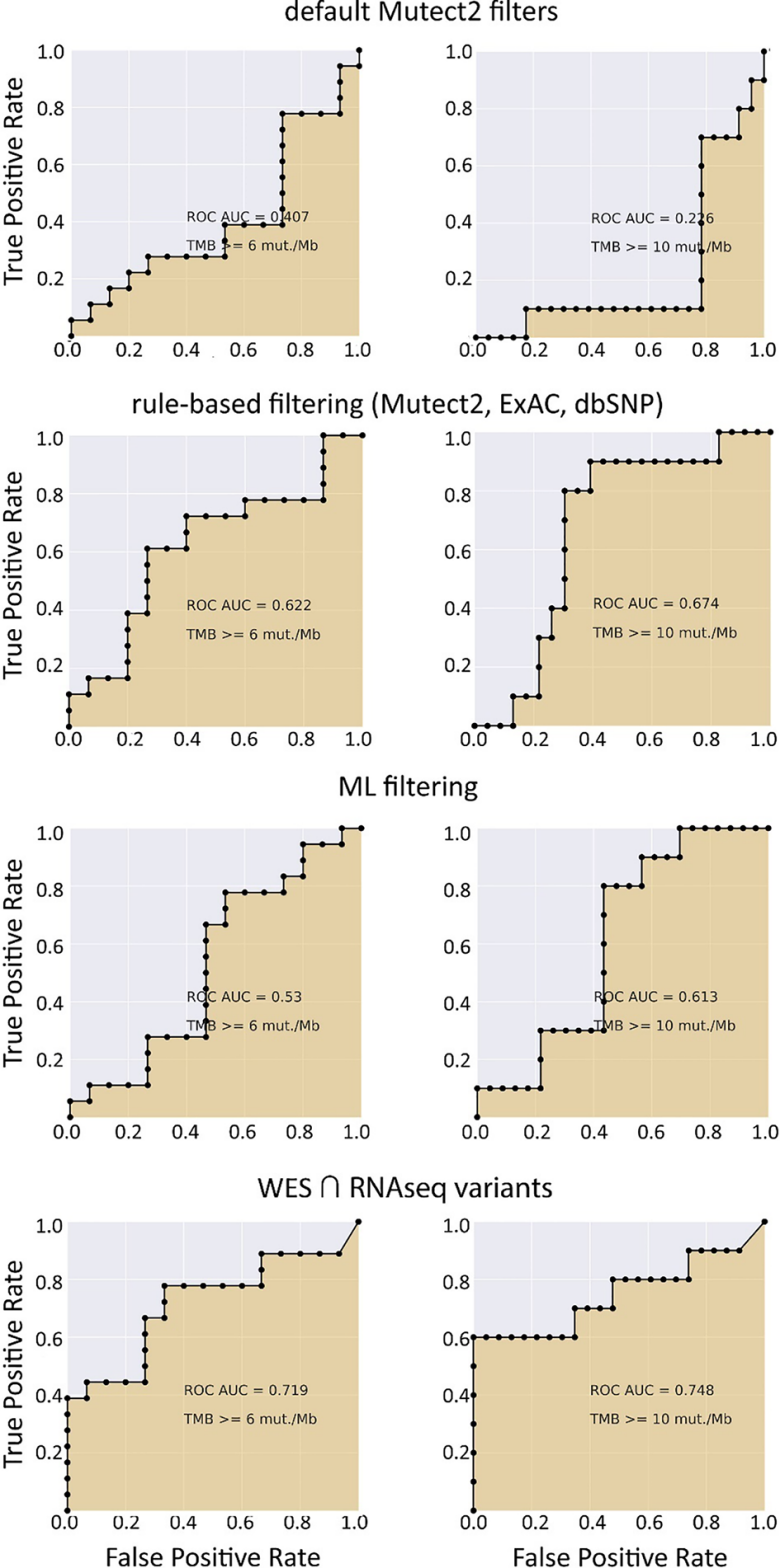
ROC AUC scores for predicting WES-TMB per megabase with RNAseq-TMB per megabase (experimental low coverage transcriptomes, *n* = 31).

We then explored the situation when RNAseq read coverage was ~2.5 times lower than that in the model TCGA dataset. In the XGBoost model, we used 34 samples as the training subset and 31 samples as the validation subset. Among the variants common to the WES and RNAseq callsets, we observed a statistically significant Pearson correlation of 0.4, *p* = 0.023. However, when applying the same rule-based filtering as with the TCGA dataset, Pearson correlation dropped to being non-significant: −0.048, *p* = 0.79 (**Figure 8**). The application of the above ML model could not significantly improve RNAseq-TMB performance and resulted in a Pearson correlation as low as 0.16 (*p* = 0.38, **Figure 8**). In all the cases, the ROC AUC scores calculated for this dataset for the thresholds of TMB >6; 10 (no significant fraction of experimental samples with TMB >20 was available, and this threshold was not characterized) were below the AUC quality threshold of 0.7 (**Figure 9** and **Table 1**).

### TMB Evaluation for Experimental RNAseq Data With “High” Coverage

**Figure 10:**
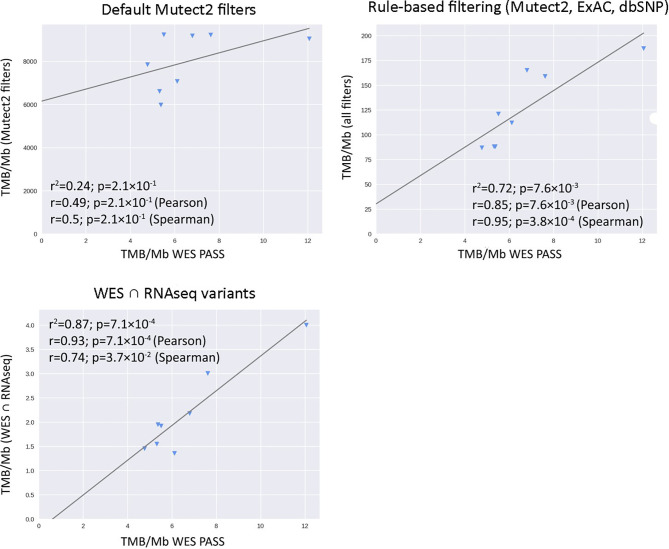
Correlations between RNAseq- and WES-derived TMB estimates in experimental high coverage RNAseq dataset, *n* = 8.

**Figure 11:**
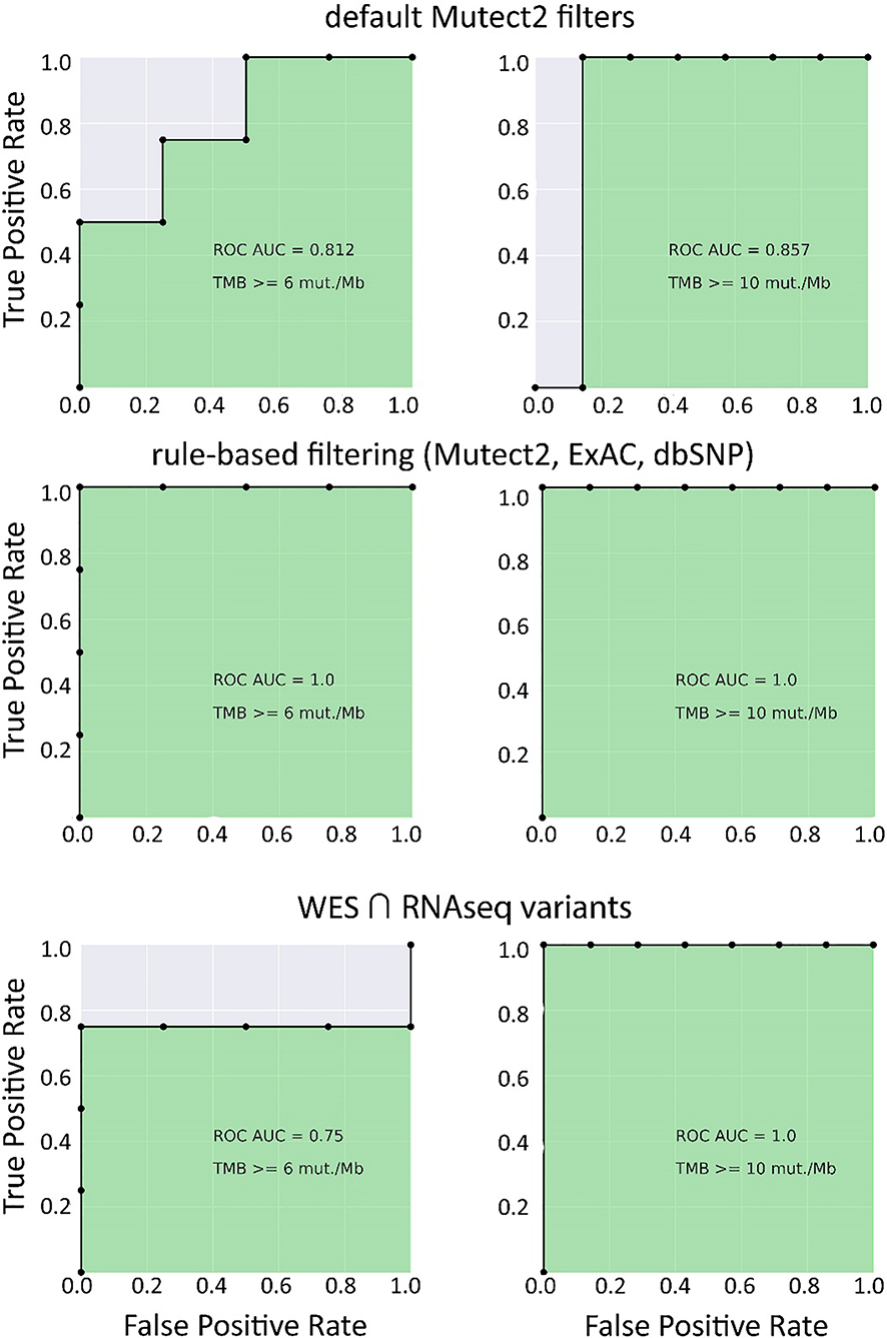
ROC AUC scores for predicting WES-TMB per megabase with RNAseq-TMB per megabase (experimental high coverage transcriptomes, *n* = 8).

In the opposite case (when RNAseq reads coverage significantly exceeded the TCGA dataset), among the variants common to the WES and RNAseq callsets, we observed a statistically significant Pearson correlation of 0.93, *p* = 7.1 * 10^−4^ (**Figure 6**). We also detected a strong correlation between the experimental RNAseq-TMB and WES-TMB estimates: Pearson correlation 0.86, *p* = 0.0056 (**Figure 10**) with AUC ~1 for TMB per megabase thresholds of >6; 10 (**Figure 11** and **Table 1**); no significant fraction of experimental samples with TMB >20 was available, and this threshold was not characterized.

Taken together, these results suggest that for the RNAseq datasets with relatively high coverage (~2.5 times higher than in TCGA), the RNAseq-TMB classifier is so strong, that no further ML-assisted improvement is needed to increase its performance.

## Discussion

Our findings suggest that RNA sequencing data for FFPE tumor tissue samples can be used to robustly assess TMB levels even in the single-sample mode. As quantified by AUC metric for the high/low TMB classification models, the performance of RNAseq-TMB estimates clearly depends on the sequencing depth. When average RNAseq depth was ~68 MGMRs, the obtained RNAseq-TMB was very well correlated with the “gold standard” WES-TMB, and the performance of the high–low binary TMB classifier was very high (AUC close to 1; **Figure 11**). In the case of mean sequencing depth of ~23 MGMRs as for the model TCGA dataset, using ML algorithms may be needed to improve the classifier robustness, thus giving the classifier AUC of ~0.8–0.9 (**Figure 4**). Finally, when the RNAseq depth is low (as ~11 MGMRs as in the low-coverage experimental dataset), the WES-TMB and RNAseq-TMB correlations are poor, and no good-quality classifier can be built even using the ML approach that used to be successful for the 23-MGMR dataset (**Figure 9**).

Our data also suggest that no healthy tissue control is needed for the FFPE-derived RNAseq data assessment to robustly estimate TMB level when the sequencing depth is sufficient (**Figure 6**).

Furthermore, WES-TMB data obtained for tumor biosamples with healthy controls correlated well with the data for the same biosamples without healthy controls (**Figure S4**). This is in line with the broad clinical practice of using targeted NGS TMB panels like FoundationOne CDx assay that does not require a healthy norm to estimate TMB (2).

Gene expression profiling by RNAseq is the alternative type of high-throughput genetic analysis that can inform clinical decision-making (51, 52). It was recently published that RNAseq data can serve as the alternative to immunohistochemical tests for several major cancer markers like HER2, ESR1, PGR, and PD-L1 (22). It can reliably estimate concentrations of cancer drug targets (53), which is also true for the emerging non-protein molecular target ganglioside GD2 (27). In addition, RNAseq data obtained for FFPE biosamples may be used to identify clinically actionable or new fusion oncogenes (26) and to generate gene signatures that can establish statuses of important tumor biomarkers like microsatellite instability (25, 54, 55) and oncogenic mutations (56) or that can predict individual sensitivity of a tumor to targeted (28, 57, 58) and non-targeted (59) therapies. Thus, adding a new option of TMB level assessment may strongly benefit this approach.

As RNAseq focuses exclusively on transcribed allele sequences, TMB calculated from such data, or another possible alternative—frequency of neoantigens, might theoretically even surpass the predictive power of WES, WGS, or target panel DNAseq with respect to the efficiency of checkpoint inhibitor immunotherapy.

Here, we provide the first experimental assessment of TMB quality for FFPE-derived RNAseq data obtained with different coverage in tumor sample-only mode. Although the results obtained are quite encouraging, the practical implementation of this technology and more detailed clinical guidelines should be a matter of further investigations with greater patient cohorts and (possibly) more specifically selected cancer types. In addition, sufficient sequencing depth threshold should be established in future studies comparing the correlation between DNA- and RNAseq-derived TMB in groups of samples with different mean coverage.

## Data Availability Statement

The datasets presented in this study can be found in online repositories. The original sequencing data were deposited to NCBI SRA with accession number PRJNA663280 allowing a free access.

## Ethics Statement

The studies involving human participants were reviewed and approved by Karelia Republic Oncological Hospital, Petrozavodsk, Russia, and Vitamed Oncological Clinical Center, Moscow, Russia. The patients/participants provided their written informed consent to participate in this study.

## Author Contributions

AB, DKu, and MSo contributed to conception and design of the study. VE, EZ, and AG organized the database and performed the ML experiments. ER, MSu, XL, and DKa organized and performed molecular analyses. MZ, VE, and AG performed the statistical analysis. MSo, EP, and AS provided the biomaterial and clinical history of the patients. AG and AB wrote the manuscript. All authors contributed to the article and approved the submitted version.

## Funding

This study was supported by the Russian Science Fund grant 20-75-10071.

## Conflict of Interest

Authors MSo, AG and AB were employed by OmicsWay Corp.

The remaining authors declare that the research was conducted in the absence of any commercial or financial relationships that could be construed as a potential conflict of interest.

## Publisher’s Note

All claims expressed in this article are solely those of the authors and do not necessarily represent those of their affiliated organizations, or those of the publisher, the editors and the reviewers. Any product that may be evaluated in this article, or claim that may be made by its manufacturer, is not guaranteed or endorsed by the publisher.

## Supplementary Material

Supplementary Figure 1 Principal component analysis (TCGA FFPE dataset). Color shows IDs of TCGA sequencing center that generated the corresponding RNAseq profile.

Supplementary Figure 2 XGBoost binary classifier feature importance scores (gain). 31 variant features were selected to train the model. 23 features are variant attributes, assigned by Mutect2. Exac_nontcga_ALL was obtained from ANNOVAR output. 7 features were engineered: Boolean features of the variant being an insertion (INS), a deletion (DEL), a transition (CT_GA) or a transversion (CA_GT)—and integer features: total depth (TOTAL_DP), as well as REF and ALT lengths (len_REF and len_ALT, respectively). See text and **Table S1** for a more detailed explanation.

Supplementary Figure 3 Precision and recall metrics for the XGBoost binary classifier predictions on the TCGA FFPE test subset, based on WES-TMB data calculated with matched normal WES profiles.

Supplementary Figure 4 TMB calculated for TCGA WXS samples called with matched normal data (x-axis) *vs* TMB calculated for TCGA WXS samples called without matched normal data (y-axis).

Supplementary Table 1 TCGA FFPE biosamples used for training and validation of XGBoost model for filtering RNAseq variants.

Supplementary Table 2 Clinical characteristics and coverage information for experimental samples.

Supplementary Table 3 Features used in training the XGBoost classifier for filtering RNAseq variant calls.
