# Supplementary figures and images for: RNA Sequencing Data for FFPE Tumor Blocks Can Be Used for Robust Estimation of Tumor Mutation Burden in Individual Biosamples
Source: Front Oncol. 2021 Sep 28;11:732644. doi: 10.3389/fonc.2021.732644 (PMC8506044; doi:10.3389/fonc.2021.732644)

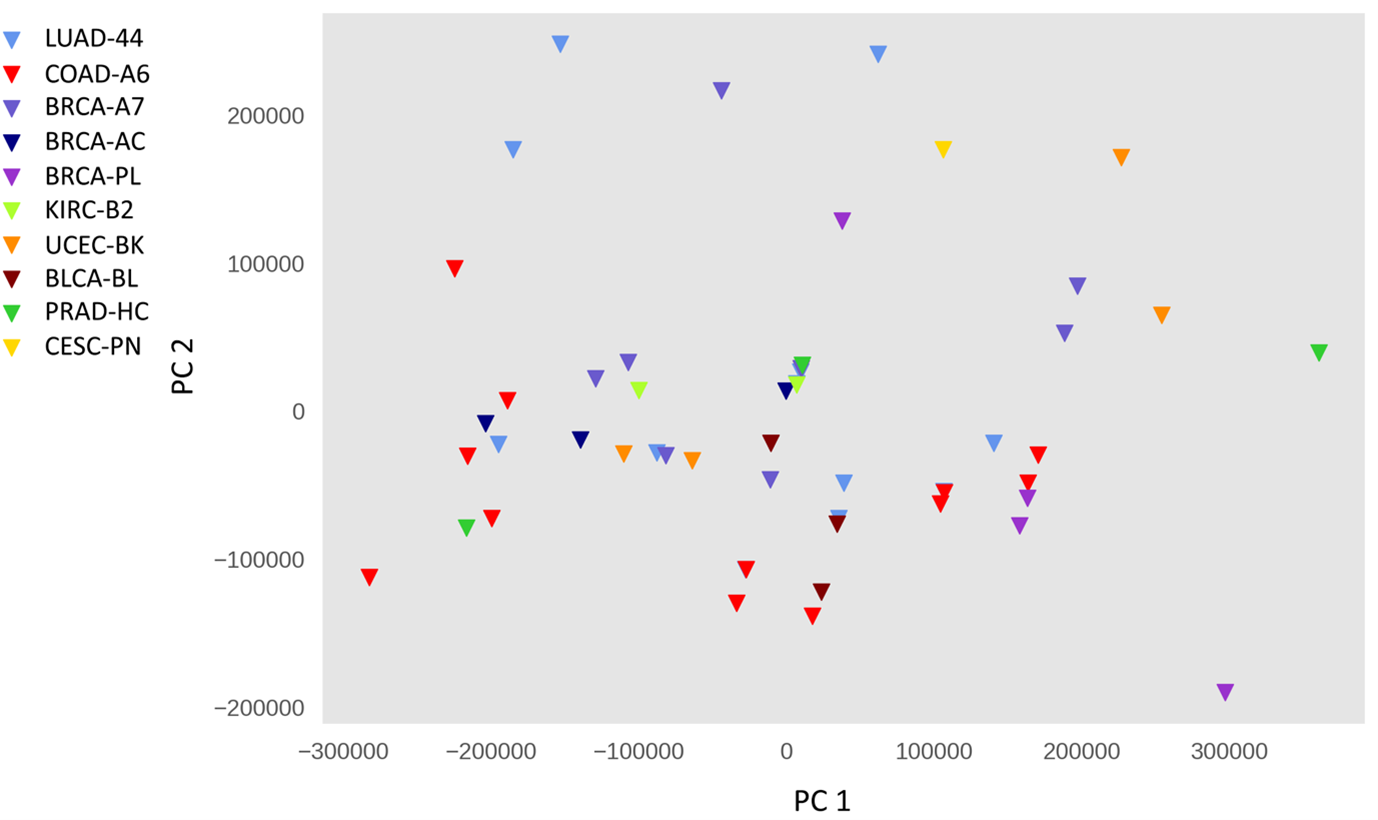

Supplement: Supplementary Figure 1 — Principal component analysis (TCGA FFPE dataset). Color shows IDs of TCGA sequencing center that generated the corresponding RNAseq profile. [file Image_1.png]

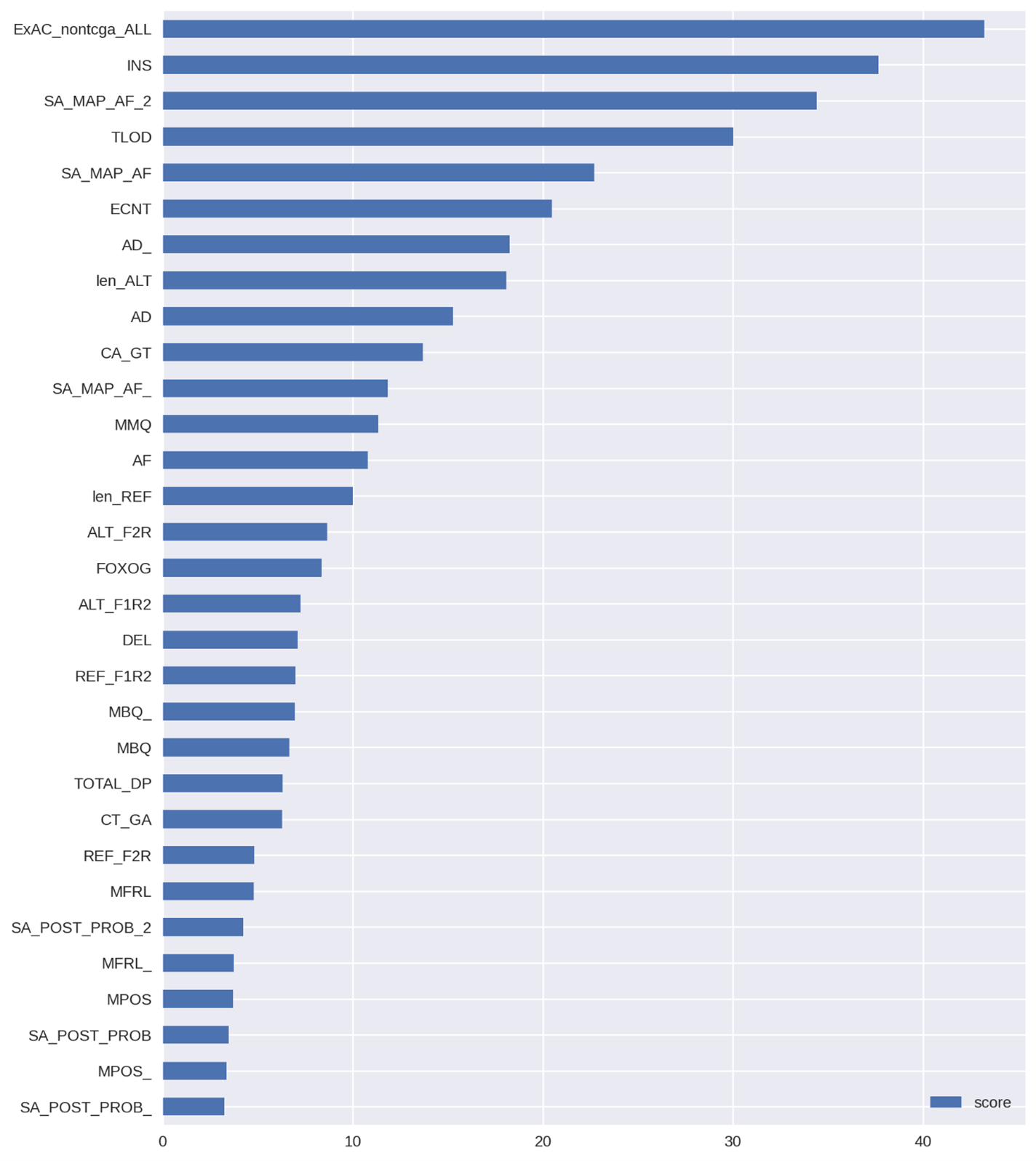

Supplement: Supplementary Figure 2 — XGBoost binary classifier feature importance scores (gain). 31 variant features were selected to train the model. 23 features are variant attributes, assigned by Mutect2. Exac_nontcga_ALL was obtained from ANNOVAR output. 7 features were engineered: Boolean features of the variant being an insertion (INS), a deletion (DEL), a transition (CT_GA) or a transversion (CA_GT)—and integer features: total depth (TOTAL_DP), as well as REF and ALT lengths (len_REF and len_ALT, respectively). See text and Table S1 for a more detailed explanation. [file Image_2.png]

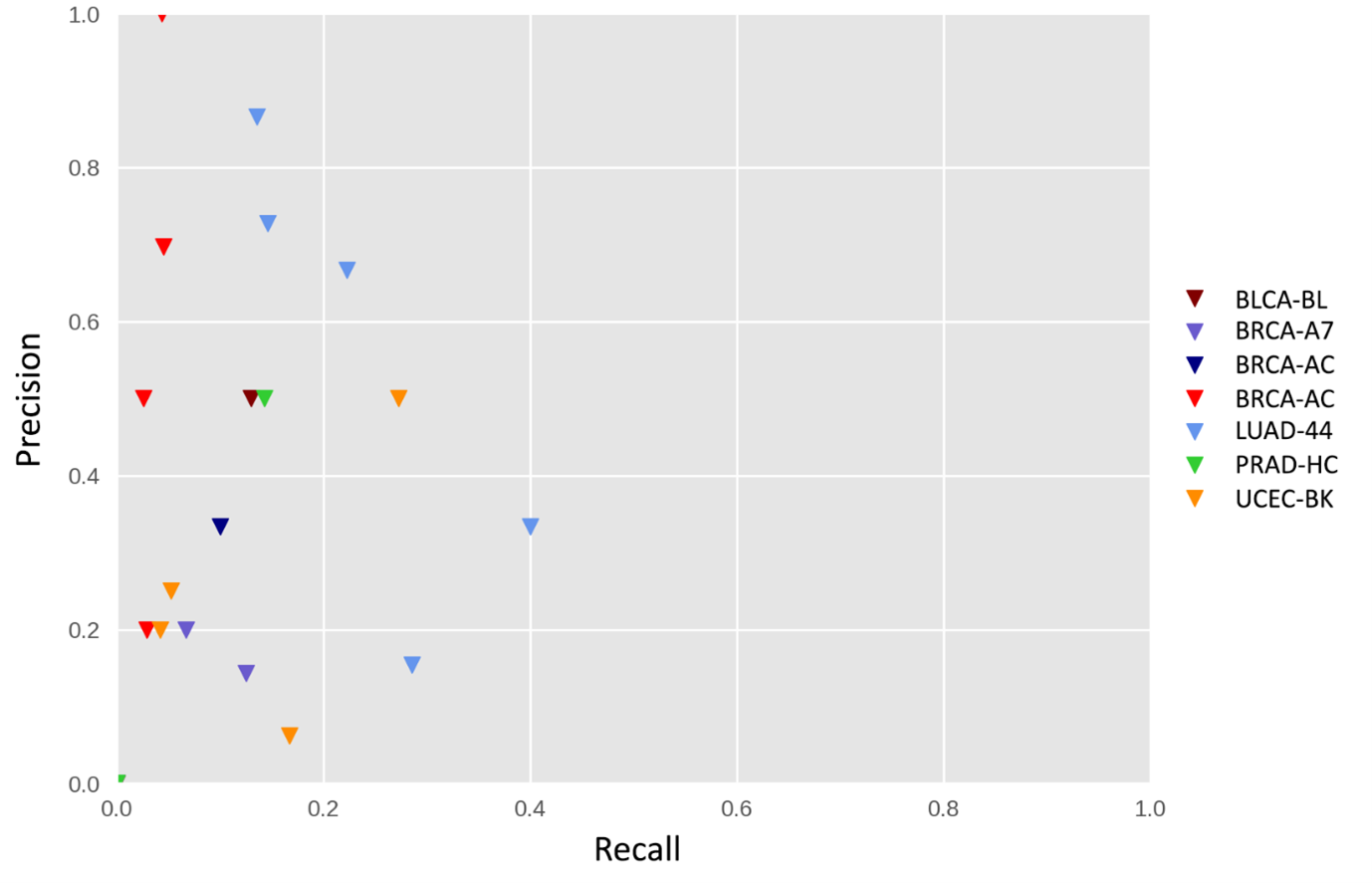

Supplement: Supplementary Figure 3 — Precision and recall metrics for the XGBoost binary classifier predictions on the TCGA FFPE test subset, based on WES-TMB data calculated with matched normal WES profiles. [file Image_3.png]

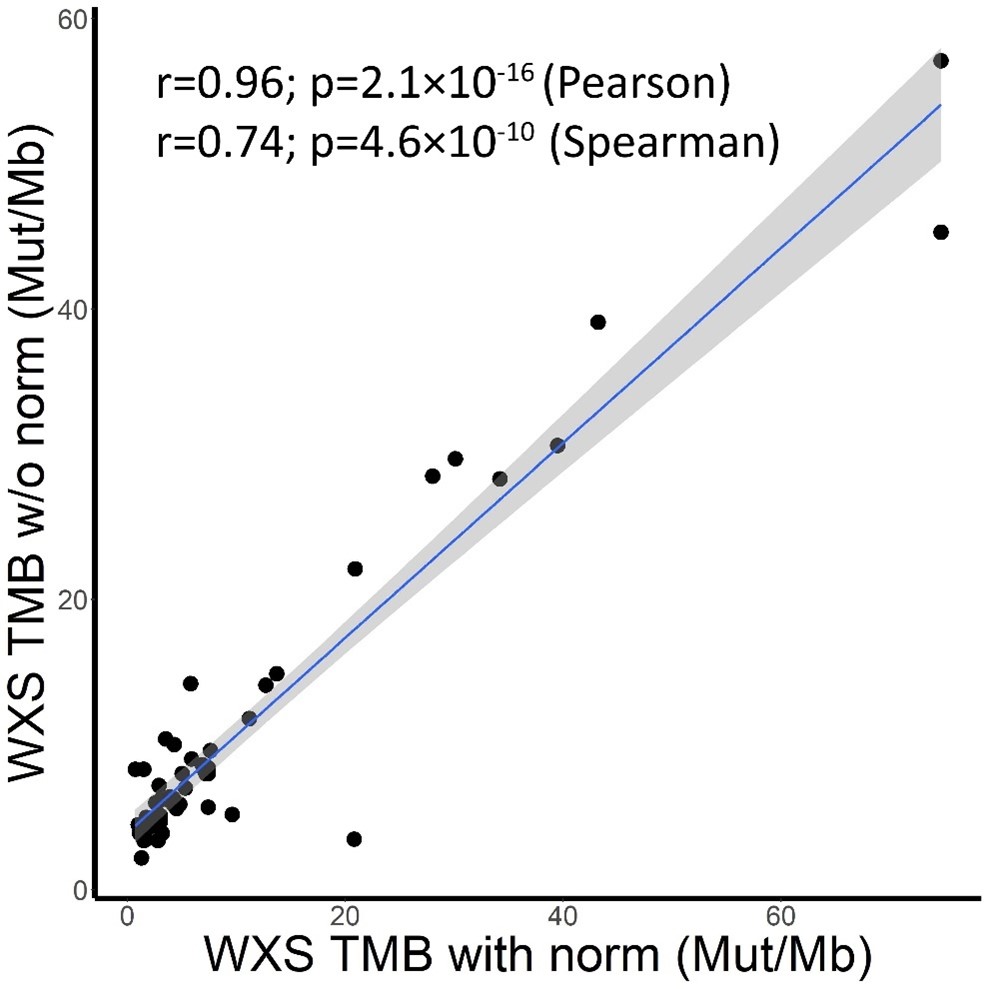

Supplement: Supplementary Figure 4 — TMB calculated for TCGA WXS samples called with matched normal data (x-axis) vs TMB calculated for TCGA WXS samples called without matched normal data (y-axis). [file Image_4.jpg]
