# Supplementary material for: RNA Sequencing Data for FFPE Tumor Blocks Can Be Used for Robust Estimation of Tumor Mutation Burden in Individual Biosamples
Source: Front Oncol. 2021 Sep 28;11:732644. doi: 10.3389/fonc.2021.732644 (PMC8506044; doi:10.3389/fonc.2021.732644)
Supplement: Supplementary Table 1 — TCGA FFPE biosamples used for training and validation of XGBoost model for filtering RNAseq variants. [file Table_1.docx]

**Table S1.** TCGA FFPE biosamples used for training and validation of XGBoost model for filtering RNAseq variants.

| **TCGA tissue code** | **Cancer type** | **Sequencing site code** | **Number of samples** |
| --- | --- | --- | --- |
| BLCA | Bladder Urothelial Carcinoma | BL | 3 |
| BRCA | Breast cancer | A7 | 8 |
| BRCA | Breast cancer | AC | 3 |
| BRCA | Breast cancer | PL | 4 |
| CESC | Cervical squamous cell carcinoma and endocervical adenocarcinoma | PN | 1 |
| COAD | Colon adenocarcinoma | A6 | 13 |
| KIRC | Kidney renal clear cell carcinoma | B2 | 2 |
| LUAD | Lung adenocarcinoma | 44 | 12 |
| PRAD | Prostate adenocarcinoma | HC | 3 |
| UCEC | Uterine Corpus Endometrial Carcinoma | BK | 4 |
