# Supplementary material for: RNA Sequencing Data for FFPE Tumor Blocks Can Be Used for Robust Estimation of Tumor Mutation Burden in Individual Biosamples
Source: Front Oncol. 2021 Sep 28;11:732644. doi: 10.3389/fonc.2021.732644 (PMC8506044; doi:10.3389/fonc.2021.732644)
Supplement: Supplementary Table 2 — Clinical characteristics and coverage information for experimental samples. [file Table_2.docx]

**Table S2.** Clinical characteristics and coverage information for experimental samples.

| Sample | Sex | Age | Cancer type | Millions of uniquely mapped reads |
| --- | --- | --- | --- | --- |
| AdC_1 | female | 43 | Adrenocortical carcinoma | 16.77 |
| AdC_2 | female | 47 | Adrenocortical carcinoma | 14.58 |
| AdC_3 | female | 43 | Adrenocortical carcinoma | 6.08 |
| BC_92 | female | 57 | Breast cancer | 7.35 |
| BC_96 | female | 55 | Breast cancer | 13.45 |
| BC_98 | female | 30 | Breast cancer | 11.73 |
| BC_99 | female | 44 | Breast cancer | 14.38 |
| BC-63 | female | 66 | Breast cancer | 10.39 |
| BC-94 | female | 35 | Breast cancer | 8.90 |
| BlC_1 | female | 72 | Bladder cancer | 7.17 |
| CC_82 | male | 57 | Colorectal cancer | 18.00 |
| CC_83 | male | 37 | Colorectal cancer | 9.34 |
| CC_87 | male | 32 | Colorectal cancer | 8.25 |
| cc-74 | female | 62 | Colorectal cancer | 13.30 |
| cc-76 | male | 60 | Colorectal cancer | 11.76 |
| cc-77 | female | 45 | Colorectal cancer | 13.01 |
| PrC_001 | male | NA | Prostate cancer | 7.65 |
| PrC_002 | male | NA | Prostate cancer | 7.38 |
| GC_25 | female | 50 | Gastric cancer | 10.49 |
| GC_26 | female | 47 | Gastric cancer | 9.20 |
| GC_28 | male | 46 | Gastric cancer | 12.82 |
| LS-4 | female | 62 | Leiomyosarcoma | 23.90 |
| LuC_27 | male | 66 | Lung cancer | 12.14 |
| LuC_44 | female | 43 | Lung cancer | 25.48 |
| LuC_46 | female | 48 | Lung cancer | 20.24 |
| LUC_47 | male | 55 | Lung cancer | 42.70 |
| LuC_49 | male | NA | Lung cancer | 15.43 |
| LuC_50 | male | NA | Lung cancer | 4.88 |
| LuC_51 | female | 51 | Lung cancer | 7.39 |
| LuC_52 | male | 59 | Lung cancer | 10.12 |
| LuC_53 | male | 43 | Lung cancer | 8.75 |
| LuC_54 | female | 56 | Lung cancer | 9.73 |
| LuC_55 | male | 65 | Lung cancer | 7.75 |
| LuC_56 | female | 60 | Lung cancer | 3.99 |
| LuC_57 | male | 48 | Lung cancer | 7.93 |
| LuC_58 | male | 46 | Lung cancer | 7.70 |
| LuC_59 | male | 71 | Lung cancer | 7.64 |
| LuC_60 | male | 69 | Lung cancer | 7.05 |
| LuC_61 | female | 44 | Lung cancer | 10.90 |
| LuC_62 | female | 55 | Lung cancer | 10.63 |
| LuC_63 | male | 58 | Lung cancer | 6.38 |
| LuC_66 | female | 82 | Lung cancer | 8.92 |
| LuC_67 | female | 53 | Lung cancer | 17.74 |
| LuC_69 | male | 70 | Lung cancer | 8.57 |
| LuC-16 | male | 75 | Lung cancer | 11.65 |
| LuC-17 | female | 52 | Lung cancer | 11.27 |
| LuC-3 | female | 51 | Lung cancer | 8.09 |
| LuC-43 | male | 64 | Lung cancer | 11.04 |
| Luc-45 | female | 61 | Lung cancer | 5.45 |
| Luc-48 | male | NA | Lung cancer | 16.94 |
| MT-2 | female | 45 | Mesothelioma | 11.17 |
| NS_13 | female | 15 | Glioblastoma | 9.63 |
| NS_15 | female | 70 | Glioblastoma | 11.79 |
| NS_16 | male | 3 | Anaplastic pleomorphic xanthoastrocytoma | 8.07 |
| NS_17 | female | 29 | Glioblastoma | 10.01 |
| NS_18 | female | 17 | Glioblastoma | 12.45 |
| NS_7 | female | 52 | Glioblastoma | 16.78 |
| OC_39 | female | 49 | Ovarian cancer | 11.14 |
| OC_40 | female | 42 | Ovarian cancer | 12.52 |
| OC_41 | female | 62 | Ovarian cancer | 10.86 |
| PC_11 | female | 71 | Pancreatic cancer | 26.71 |
| PC_13 | female | 51 | Pancreatic cancer | 15.26 |
| PC_17 | female | 68 | Pancreatic cancer | 10.70 |
| PC_18 | male | 49 | Pancreatic cancer | 6.74 |
| PrC_3 | male | 66 | Prostate cancer | 8.94 |
| CC_14 | female | 65 | Colorectal cancer | 78.94 |
| CC_16 | male | 76 | Colorectal cancer | 78.94 |
| E_3 | male | 47 | Normal esophagus | 52.72 |
| CC_9 | male | 76 | Colorectal cancer | 49.48 |
| BC_72 | female | 79 | Breast cancer | 82.18 |
| LuC_18 | male | 63 | Lung cancer | 62.33 |
| LuC_35 | male | 75 | Lung cancer | 70.16 |
| LuC_37 | male | 68 | Lung cancer | 66.65 |
