# Supplementary material for: RNA Sequencing Data for FFPE Tumor Blocks Can Be Used for Robust Estimation of Tumor Mutation Burden in Individual Biosamples
Source: Front Oncol. 2021 Sep 28;11:732644. doi: 10.3389/fonc.2021.732644 (PMC8506044; doi:10.3389/fonc.2021.732644)
Supplement: Supplementary Table 3 — Features used in training the XGBoost classifier for filtering RNAseq variant calls. [file Table_3.docx]

**Table S3.** Features used in training the XGBoost classifier for filtering RNAseq variant calls.

| **Feature** | **Source** | **Type** | **Description** |
| --- | --- | --- | --- |
| ExAC_nontcga_ALL | ANNOVAR | real | overall frequency of the allele observed in the ExAC database, excluding participants from the TCGA project |
| SA_MAP_AF(_1, _2) | Mutect2 | real | the maximum likelihood estimates of the allele fraction given an artifact on the forward strand, reverse strand, or no artifact |
| INS | Engineered | Boolean | variant is an insertion |
| TLOD | Mutect2 | real | tumor LOD: Log odds that the variant is present in the tumor sample relative to the expected noise |
| ECNT | Mutect2 | integer | number of events in the given haplotype |
| AD(_1) | Mutect2 | integer | allelic depths for the reference and alternative alleles |
| len_ALT | Engineered | integer | length of the alternative allele |
| CA_GT | Engineered | Boolean | variant is a transversion |
| AF(_1) | Mutect2 | real | allele frequency for each ALT allele |
| MMQ | Mutect2 | integer | median mapping quality of each alternate allele |
| len_REF | Engineered | integer | length of the reference allele |
| FOXOG | Mutect2 | real | the fraction of alt reads indicating OxoG error, induced by DNA oxidation during library preparation |
| ALT_F2R1 | Mutect2 | integer | the number of reads in the F2R1 orientation supporting the alternate allele |
| REF_F1R2 | Mutect2 | integer | the number of reads in the F1R2 orientation supporting the reference allele |
| ALT_F1R2 | Mutect2 | integer | the number of reads in the F1R2 orientation supporting the alternate allele |
| DEL | Engineered | Boolean | variant is a deletion |
| CT_GA | Engineered | Boolean | variant is a transition |
| TOTAL_DP | Engineered | integer | sum of read depths for reference and alternative allele |
| REF_F2R1 | Mutect2 | integer | the number of reads in the F2R1 orientation supporting the reference allele |
| MFRL(_1) | Mutect2 | integer | median fragment length of reads supporting each allele |
| MPOS(_1) | Mutect2 | integer | median distance from the end of the read for each alternate allele |
| SA_POST_PROB(_1) | Mutect2 | real | the normalized posterior probability that there is an artifact on the forward strand, reverse strand, or no artifact |
